# Spectroscopic Parameter and Molecular Constant Investigations on Low-Lying States of BeF Radical

**DOI:** 10.3390/ijms13022501

**Published:** 2012-02-22

**Authors:** Zun Lue Zhu, Qing Peng Song, Su Hua Kou, Jian Hua Lang, Jin Feng Sun

**Affiliations:** College of Physics & Information Engineering, Henan Normal University, Xinxiang 453007, China; E-Mails: shangen1987@163.com (Q.P.S.); coco13141@126.com (S.H.K.); langjianhua87@163.com (J.H.L.); jfsun@htu.cn (J.F.S.)

**Keywords:** potential energy curve, dissociation energy, spectroscopic constant, molecular constant

## Abstract

The potential energy curves (PECs) of X^2^∑^+^, A^2^Π_r_ and B^2^∑^+^ states of BeF radical have been investigated using the complete active space self-consistent-field (CASSCF) method, followed by the highly accurate valence internally contracted multireference configuration interaction (MRCI) approach at the correlation-consistent basis sets, cc-pV5Z for Be and aug-cc-pV6Z for F. Based on the PECs of X^2^∑^+^, A^2^Π_r_ and B^2^∑^+^ states, the spectroscopic parameters (*D_e_*, *R_e_*, *ω_e_*, *ω_e_χ_e_*, *α_e_* and *B_e_*) have also been determined in the present work. With the PECs determined at the present level of theory, vibrational states have been predicted for each state when the rotational quantum number *J* equals zero (*J* = 0). The vibrational levels, inertial rotation and centrifugal distortion constants are determined for the three states, and the classical turning points are also calculated for the X^2^∑^+^ state. Compared with the available experiments and other theories, it can be seen that the present spectroscopic parameter and molecular constant results are more fully in agreement with the experimental findings.

## 1. Introduction

Fluorides are a very important chemical species with broad applications in chemistry. The chemical property of fluorine is very lively and highly oxidized. In combination with other elements, resultant properties will be heat-resistant and difficult to erode by drugs and solvents. Fluorine is widely used in domestic appliances, office automation equipment, semiconductors, automobiles and other fields. Recently, with the development of calculation technology of quantum chemistry, more and more interest has been concentrated on the beryllium compounds [1–6]. As a simple fluoride compound, Beryllium Monofluoride (BeF) has been widely studied, both experimentally [7–11] and theoretically [12–21].

However, as can be seen in the literature, the experimental dissociation energies *D*_0_ of BeF greatly differ from each other. For example, the value reported by Hildenbrand and Murad [7] in 1966 is of 5.85 eV and the value determined by Farber and Srivastava [9] in 1974 is of 6.26 eV. Whereas this value collected in Reference [10] by Herzberg in 1950 is of 5.4 eV and collected in Reference [11] by Huber and Herzberg in 1979 is of 6.26 or 5.85 eV. Obviously, it needs to be clarified urgently.

In theory, the spectroscopic parameters including the dissociation energy *D_e_* have been widely studied in the past several decades [12–21]. On the one hand, the *D_e_* values still show a wide variation. For example, Roach and Kuntz [12] investigated the *D_e_* in 1982, and gave a value of 3.94 eV. Partridge *et al.* [13] calculated the *D_e_* in 1984 with a value of 5.94 eV. On the other hand, it is still in question whether the potential barrier on the ground-state potential energy curve exists or not. For example, Roach [12] and Machado *et al.* [17] thought that the barrier obtained here, and the spectroscopic parameters are accurately determined. Finally, it is considered that numerically solving the radial Schrödinger equation is possible, but Marian [14] and Ornellas *et al.* [18] did not think so. Furthermore, some theoretical information [14,18,20,21] is available about the excited states of BeF. Some vibrational manifolds (such as vibrational levels, initial rotation and centrifugal distortion constants) have been reported in the literature, which have important applications in the vibrational transition calculations. All these aspects motivated us to perform the present investigations.

One of the purposes of this investigation is to determine the accurate potential energy curves of X^2^∑^+^, A^2^Π_r_ and B^2^∑^+^ states for BeF radical, using the full valence complete active space self-consistent field method [22,23], followed by the highly accurate valence internally contracted multireference configuration interaction approach [24,25] in combination with the correlation-consistent basis sets [26–28], cc-pV5Z for Be and aug-cc-pV6Z for F atom. The spectroscopic parameters and vibrational manifolds are determined for these three states, using the obtained PECs of BeF radical, with the help of VIBROT module in MOLCAS 7.4 program package [29].

## 2. Theoretical Approach

We calculate the PECs of X^2^∑^+^, A^2^Π_r_ and B^2^∑^+^ states of BeF by the CASSCF approach, followed by the MRCI calculations. Therefore, the full valence CASSCF is employed as the reference wavefunction for the MRCI calculations in the present work. For the PEC calculations, the MRCI theory has proven particularly successful [30–35]. The present calculations are carried out in MOLPRO 2008.1 program package [36] with the largest correlation-consistent basis set, cc-pV5Z for Be and aug-cc-pV6Z for F atom.

BeF is of C_∞_*_v_* point group symmetry. According to the molecular theory and the requirement of MOLPRO program package, it must be replaced by C_2_*_v_* symmetry with the order of the irreducible representations as *a*_1_/*b*_1_/*b*_2_/*a*_2_ in the calculations. In detail, eight molecular orbitals (MOs) are put into the active space, including four *a*_1_, two *b*_1_ and two *b*_2_ symmetry MOs, which correspond to the 2*s* shell of Be and 2*s*2*p* shell of F atom. The rest of the electrons in the BeF radical are put into the closed-shell orbitals, including two *a*_1_ symmetry MOs. When we use these MOs (six *a*_1_, two *b*_1_, two *b*_2_) to calculate the PECs of the BeF radical, we find that the obtained PECs are smooth for all these basis sets over the present internuclear distance range.

In general, the PECs calculations are made at intervals of 0.02 nm over the internuclear distance range from 0.0522 to 2.0472 nm. Near the equilibrium position, we chose the interval to be of 0.005 nm so that the properties of the PECs are displayed more clearly. With the PECs determined at the different basis sets, the spectroscopic parameters (*D_e_*, *ω_e_*, *ω_e_χ_e_*, *α_e_*, *B_e_* and *D*_0_) are evaluated. By comparison with the experiments [7–11], we find that the best favorable spectroscopic parameter results can be obtained at the basis sets, cc-pV5Z for Be and aug-cc-pV6Z for F atom.

In order to take into consideration the relativistic effects on the spectroscopic parameters, the Douglas-Kroll one-electron integrals are used with the basis sets cc-pV5Z for Be and aug-cc-pV6Z for F. We notice that almost no accuracy improvements can be made for the spectroscopic parameters after considering the relativistic corrections. Therefore, vibrational manifold calculations are made at the PECs obtained at the non-relativistic condition.

## 3. Results and Discussion

### 3.1. PECs of the BeF and Spectroscopic Parameters

**Figure 1:**
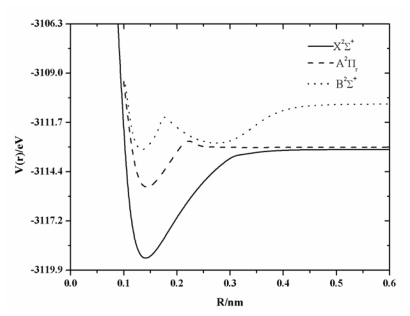
Potential energy curves (PECs) of the BeF.

**Figure 2:**
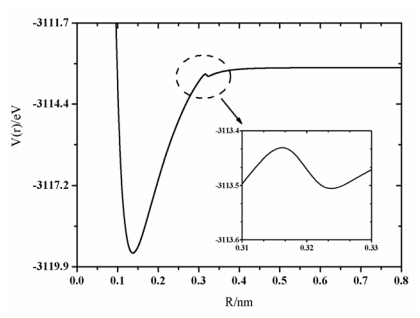
PEC of the X^2^∑^+^state.

The PECs of BeF radical are shown in Figure 1. As shown in the figure, the A^2^Π_r_ curve and the B^2^∑^+^ curve are all marginally repulsive at long range, but they do not converge. The A^2^Π_r_ state and the X^2^∑^+^ state have the same dissociation channel Be(^1^S_g_) +F(^2^P_u_), which is different from Be(^3^P_u_) +F(^2^P_u_) for the B^2^∑^+^ state. During the course of the PEC investigation of the X^2^∑^+^ state, the existence of the barrier was a hot topic and should be stressed here, however, that it is not the main goal of the present work. To illustrate the existence of the barrier of the PEC of the X^2^∑^+^ state, a magnified image for the PEC of the X^2^∑^+^ state has been shown in Figure 2. It has been found in our calculations that there is a small barrier in the curve of X^2^∑^+^ state which has been found at the internuclear separation, 0.3372 nm, and the barrier height is of 0.18 eV. A similar situation was also found by Roach [12] and Machado [17], but not by Marian [14] and Ornellas *et al.* [18]. Ornellas *et al.* [18] did not observe the small hump since the interval used was too large when they calculated the PEC. Marian [14] paid attention to calculating the spin-orbit coupling, and he considered 42 reference state functions to generate the CI wavefunction. In similarity with Reference [18], the interval was also too large in his calculations [14]. A wide barrier of 0.79 eV has been found in the PEC of the A^2^Π_r_ state, similar to the value reported by Marian [14] and Ornellas *et al.* [18], 0.81 eV and 0.79 eV, respectively. A similar feature has also been found for the B^2^∑^+^ curve of the BeF radical. Near 0.18nm, the B^2^∑^+^ state unfolds a sharp avoided crossing with the repulsive covalent state correlating with the dissociation channel Be(^3^P_u_) +F(^2^P_u_). So the avoided crossing and the ionic character are responsible for the unusual shape of these potential curves.

With the PECs determined, the spectroscopic parameters and molecular constants are evaluated with the VIBROT module in MOLCAS 7.4 program package. In order to conveniently compare the present results, we compiled the spectroscopic parameters together with the available experiments [7–11] and other theories [12–21] in Table 1 for the BeF radical.

A number of theoretical investigations had been made on the spectroscopic parameters of the X^2^∑^+^ state of the BeF radical. Partridge *et al.* [13] in 1984 carried out the *R_e_*, *D_e_* and *D*_0_ calculations using Hartree-Fock (HF) method and some empirical formulas with Slater-type orbital (STO) basis set. Although their calculational results are close to the experiments, the existing experimental values and some empirical formulas were used and only two spectroscopic parameters were evaluated in their investigations. In 1985, Marian [14] investigated the PEC using multireference doubles configuration interaction approach (MRDCI) method with the GTO DZP AO basis set. With the aid of PEC, they calculated several spectroscopic parameters. We can find that his *ω_e_χ_e_* is slightly smaller than the present one when compared with the corresponding experiments, though his *R_e_* is in more agreement with the experiments than ours. Langhoff *et al.* [15] in 1986 calculated *R_e_* and *ω_e_* by two methods. We find that their most favorable results were obtained by the configuration interaction (CI) approach. As shown in Table 1, it is believed that these results are the most accurate values so far, but only limited spectroscopic parameters are derived. Langhoff *et al.* [16] later evaluated the *R_e_* and *ω_e_* by three approaches. By comparison with the experiments, we find that their most favorable results were obtained with the singles and doubles configuration interaction (SDCI) approach. Also, the values are in more agreement with the experiments when compared with the present ones. However, their investigations were not concerned with other spectroscopic parameters.

Later, Machado and Ornellas [17] in 1989 made the PEC calculations by multireference singles and doubles configuration interaction approach (MRSDCI) with the Gaussian sets (5*s*, 3*p*) for Be and (7*s*, 4*p*) for F. As can be seen in Table 1, their *ω_e_* and *ω_e_χ_e_* are too large when compared with the experiments. Three years later, Ornellas *et al.* [18] in 1992 made the PEC calculation for ground state. In the calculations, their approach is the MRSDCI and the basis sets are (14*s*10*p*3*d*1*f*)/[8*s*6*p*3*d*1*f*] for F and (11*s*6*p*1*d*)/[6*s*4*p*1*d*] for Be. By comparison with the present ones, it is not difficult to find that their *ω_e_χ_e_* and *ω_e_* are slightly larger than the present experiments. Recently, Li and Hamilton [19] in 2001 calculated the *R_e_* using density functional theory (DFT) and MØller-Plesset (MP2) methods with three basis sets. Their most favorable results were obtained by DFT (BH and HLYP) approach with 6 − 311 + G* basis sets. However, they did not compute spectroscopic parameters apart from the *R_e_* and *ω_e_*. Recently, Pelegrini *et al.* [20] in 2005 performed some spectroscopic parameter calculations by the MRCI method with the aug-cc-pVQZ basis set. As tabulated in Table 1, their *ω_e_χ_e_* is far from the measurements when compared with the present work. Furthermore, other important spectroscopic parameters (such as *B_e_* and *α_e_*) were not evaluated in their investigations.

For the A^2^Π_r_ state, Walker and Richards [21] performed the *R_e_* and *ω_e_* calculations using two methods in 1967. We find that their optimal results were obtained by the configuration interaction (CI) approach. As shown in Table 1, their *ω_e_* is slightly smaller than the experiment data and other important spectroscopic parameters were not evaluated in their investigations. In 1985, Marian [14] investigated the PEC using MRDCI method with a GTO DZP AO basis set, with the aid of PEC, they calculated several spectroscopic parameters. We can find that his *ω_e_χ_e_* is too large and his *D*_e_ is too small when compared with the experiments. Furthermore, *α_e_* was not evaluated in his investigations. Ornellas *et al.* [18] in 1992 made the PEC calculation for lowest-lying state. In the calculations, their approach is the MRSDCI and the basis sets are (14*s*10*p*3*d*1*f*)/[8*s*6*p*3*d*1*f*] for F and (11*s*6*p*1*d*)/[6*s*4*p*1*d*] for Be. By comparison, it is not difficult to find that their *ω_e_χ_e_* and *ω_e_* are slightly larger than the present experiments when compared with the present ones. Pelegrini *et al.* [20] also performed some spectroscopic parameter calculations for the A^2^Π_r_ state of the BeF radical using the MRCI method with the aug-cc-pVQZ basis set. As tabulated in Table 1, their *ω_e_χ_e_* and *ω_e_* are far from the available measurements when compared with our work.

For the B^2^∑^+^ of BeF radical, few theoretical investigations have been made on the spectroscopic parameters. The earlier theoretical calculations were performed by Marian [14]. He investigated the PEC of BeF(B^2^∑^+^) using MRDCI method with a GTO DZP AO basis set. We can find that his *ω_e_* and *ω_e_χ_e_* are too large when compared with the experiments. Furthermore, *D*_e_ and *α_e_* were not evaluated in his investigations.

According to the above analysis and discussion, on the whole, the spectroscopic parameters obtained in the present work have improved when compared with previous theoretical results. For example, for the X^2^∑^+^ state, the spectroscopic parameters, *ω_e_χ_e_*, *α_e,_* *ω_e_*, *B_e_* and *R*_e_, deviate from the experiments [11] only by 0.11%, 0.57%, 0.90%, 1.60% and 0.81%, respectively. For the BeF(A^2^Π_r_), the spectroscopic parameters, *ω_e_χ_e_*, *α_e,_* *ω_e_*, *B_e_* and *R*_e_, deviate from the experiments [11] only by 0.00%, 2.86%, 1.69%, 0.51% and 0.25%, respectively.

As for the dissociation energy *D_e_* of BeF(X^2^∑^+^), it shows a wide variation. Roach and Kuntz [12] in 1982 made valence-bond (VB) calculations on the BeF(X^2^∑^+^) radical, and they obtained the value to be 3.94 eV. But they claimed that their VB calculations are not accurate enough to deduce the accurate value of *D_e_* in Reference [12]. Partridge *et al.* [13] calculated the *D*_0_ with empirical formula and obtained the direct value of *D*_0_ to be 5.86 eV, and also gave the estimate result of 5.91 eV. The precision of the method is slightly lower than this work. Marian [14] investigated the PEC using MRDCI method with a GTO DZP AO basis set. They obtained *D_e_* of 5.5 eV, however, he thought that the value is a little small. Langhoff *et al.* [15] calculated the *D*_e_ by the SCF method. As we know, the method is too simple so that the *D_e_* result they obtained is not very credible. Machado and Ornellas [17] calculated the *D*_e_ by MRSDCI approach with the Gaussian sets (5s,3p) for Be and (7s,4p) for F. Ornellas *et al.* [18] computed the *D*_e_ by the MRSDCI method and the basis sets are (11s6p1d)/[6s4p1d] for Be and (14*s*10*p*3*d*1*f*)/[8*s*6*p*3*d*1*f*] for F. The basis sets they used are very small. Therefore, their values are less accurate. In the present work, the PEC of BeF(X^2^∑^+^) is computed using the highly accurate MRCI approach with the large basis sets, cc-pV5Z for Be and aug-cc-pV6Z for F. With the aid of PEC, the *D_e_* is determined to be 6.22 eV, which should be relatively close to the true value.

In this paper, we also calculate the *ΔT_e_* of the A^2^Π_r_ state is of 32,343.9 cm^−1^, while the value obtained by Marian [14], Ornellas *et al.* [18] and Pelegrini *et al.* [20] to be 34,814 cm^−1^, 33,974 cm^−1^ and 34,902 cm^−1^, respectively. And the *ΔT_e_* of the B^2^∑^+^ state is also calculated, and the value is of 48,877 cm^−1^, the data reported by Marian [14] to be 50,844 cm^−1^.

It is widely recognized that the accuracy of the spectroscopic parameters calculations mainly depends on the scanned results for the PEC of the electronic state by using CASSCF AND MRCI approach. The scanned results of the electronic state are related to the choice of the active space for a CASSCF and of the basis sets. For BeF radical, the each electronic state possesses different bonding orbitals at various internuclear sparations [14]. In order to obtain more accurate calculational results of PECS of BeF radical, eight molecular orbitals, including four *a*_1_, two *b*_1_ and two *b*_2_ symmetry MOs, are put into the active space, and the rest of the electrons in the BeF radical are put into two *a*_1_ symmetry closed-shell orbitals, which differ from Reference [20]. In addition, the appropriate choices of the basis sets and the calculational interval in the CASSCF calculation also conduce to the accurate calculational results. So we have reasons to believe that the present results are reliable.

### 3.2. Vibrational Manifolds

Based on the reliable PECs of the X^2^∑^+^, A^2^Π_r_ and B^2^∑^+^ states, we determine their vibrational levels, inertial rotation and centrifugal constants when *J* = 0. And we also compute classical turning points for the ground state. Owing to the length limitation of the paper, we only tabulate some of these results for the vibrational states in Tables 2–7. To the best of our knowledge, no experimental data of molecular constants have been found in the literature, except several groups of theoretical results. But according to the remarkable agreement between the present spectroscopic parameters and the available experiments and the excellent accordance between the theoretical and the corresponding RKR data, we have reasons to believe that the results collected in Tables 2–7 are accurate.

As can be seen from Table 2, the present results are in excellent agreement with the theoretical data reported in the literature. For example, the deviations from the theories [17] are of only 0.25%, 0.12%, 0.02% and 0.23% when *υ* = 1, 3, 5 and 7, respectively, and the deviations from the theories [18] deviate only by 0.23%, 0.33%, 0.45% and 0.64%, respectively. Therefore, we can say that the present calculations are accurate. Furthermore we can conclude that the values of vibrational levels and classical turning points presented in Table 3 must be reliable.

Similar to the vibrational level spacings, there are two groups of theoretical data [17,18] concerned with the inertial rotation constant *B_υ_* and centrifugal distortion constant *D_υ_* of BeF(X^2^∑^+^). For a convenient comparison with the present results, we also tabulate them in Table 4. By simple calculations, it is not difficult to find that excellent agreement exists between the present results and the theoretical data. For example for the *B_υ_*, the deviations from the theory [17] are only 0.14%, 0.47%, and 0.51% when *υ* =0, 2 and 4, respectively. As to the centrifugal distortion constant *D_υ_*, good accord also exists between the present results and the available theoretical data [17,18]. Therefore, the present calculations are accurate. According to these, the calculations of the centrifugal distortion constants presented in Table 5 should be reliable.

As can be seen from Table 6, the present results are in excellent agreement with the experiments [14]. For example, the deviations from the experiments [14] are only 0.13%, 0.19%, 0.27% and 0.38% when *υ* = 0, 2, 4 and 6, respectively. Therefore, we can say that the present calculations are accurate. For the inertial rotation constant *B_υ_*, the deviations of the present values from the experiments [8] are of 0.50% and 0.45%, when *υ* = 0 and 1, respectively.

To the best of our knowledge, no experimental and theoretical data of vibrational levels and molecular constants for BeF(B^2^∑^+^) has been found in the literature. However, according to the remarkable agreement between the present spectroscopic parameters and the available experiments [8,11], we have reasons to believe that the results collected in Tables 5 are accurate.

## 4. Conclusions

In the present work, the PECs of X^2^∑^+^, A^2^Π_r_ and B^2^∑^+^ states of BeF radical have been investigated by the MRCI approach with large correlation-consistent basis sets, cc-pV5Z for Be and aug-cc-pV6Z for F. Based on the PECs of these three states, the spectroscopic parameters and molecular constants are determined in the present work, and the values are in excellent agreement with the experimental data. With the PECs of these states determined at the MRCI level of theory, the vibrational levels, inertial rotation and centrifugal distortion constants are predicted, and the classical turning points are also calculated for the X^2^∑^+^ state when *J* = 0. On the whole, comparison with the available experiments and theories shows that the present calculations are both reliable and accurate.

## Figures and Tables

**Table 1:** Spectroscopic parameter comparison with available measurements and other theories for BeF radical.

Source	*D_e_*/eV	*R_e_*/nm	*ω_e_*/cm^−1^	*ω_e_χ_e_*/cm^−1^	*B_e_*/cm^−1^	*α_e_*/cm^−1^	*D*_0_/eV
**X^2^∑^+^**							
This work	6.22	0.1372	1236.12	9.11	1.4651	0.0175	6.14
Exp [7]	----	----	----	----	----	----	5.85
Exp [9]	----	----	----	----	----	----	6.26
Exp [10]	5.48	0.13614	1265.6	9.12	1.4877	0.01685	5.4
Exp [11]	6.34 or 5.93	0.1361	1247.36	9.12	1.4889	0.0176	6.26 or 5.85
Theory [12]	3.94	----	----	----	----	----	----
Theory [13]	5.94	0.135	----	----	----	----	5.86
Theory [14]	5.5	0.1369	1258	8.8	1.472	----	----
Theory [15]							
SCF	----	0.1352	1280	----	----	----	5.88
CI(SD)	----	0.1363	1250	----	----	----	5.94
Theory [16]	----	0.13637	1250	----	----	----	----
Theory [17]	6.00	0.13711	1265.7	9.26	1.469	0.0169	5.92
Theory [18]	5.82	0.1369	1272.5	9.52	1.472	0.01695	----
Theory [19]	----	0.137	1240	----	----	----	----
Theory [20]	----	0.13531	1339.3	8.34	----	----	----
**A^2^Π_r_**							*T*_e_/cm^−1^
This work	2.32	0.1397	1174.2	8.78	1.413	0.0170	32,343.9
Exp [8]	----	0.13935	1171.2	-----	1.42024	0.0175	33,187
Exp [10]	----	0.13941	1172.6	8.78	1.4186	0.0161	33,233.6
Exp [11]	1.81 or 2.22	0.13935	1154.67	8.78	1.42024	0.0175	33,233.6
Theory [14]	1.17	0.1387	1183	13.5	1.433	----	34,814
Theory [18]	1.69	0.1395	1175.4	8.8	1.412	0.01713	33,974
Theory [20]	----	0.1385	1226.8	7.42	----	----	34,902
Theory [21]	----	0.1437	1116	----	----	----	----
**B^2^∑^+^**							
This work	2.60	0.1332	1351.1	12.7	1.554	0.0149	48,877
Exp [8]	----	0.1335	1350.8	----	1.547	----	49,573
Exp [11]	2.51 or 2.977	0.1335	1350.8	12.6	1.547	----	49,570
Theory [14]	----	0.1321	1503	13.1	1.580	----	50,844

**Table 2:** Comparison of the present and other theoretical vibrational level spacings (in cm**^−^**^1^), *G*(*υ* + 1) − *G*(*υ*).

*υ*	This work	Ref. [17]	Ref. [8]	Ref. [18]	*υ*	This work	Ref. [17]	Ref. [8]	Ref. [18]
0	1254.0	1255.6	1254.5	1247.2	14	1021.1	1024.4	1009.3	1003.7
1	1236.4	1239.5	1233.6	1229.0	15	1005.4	1007.7	993.0	987.4
2	1218.9	1221.6	1215.4	1210.8	16	989.8	991.5	997.0	
3	1201.5	1202.9	1197.5	1192.8	17	947.3	975.7	961.4	
4	1184.5	1184.8	1179.7	1175.0	18	958.8	960.4		
5	1167.5	1167.7	1162.3	1157.4	19	943.5	945.6		
6	1150.7	1151.9	1144.5	1139.5	20	928.2	931.3		
7	1134.0	1136.6	1126.8	1122.2	21	912.9	917.5		
8	1117.5	1121.4	1109.4	1104.9	22	897.8	904.0		
9	1101.2	1106.2	1092.1	1086.8	23	882.6	890.8		
10	1084.9	1090.6	1075.1	1070.6	24	867.5	877.8		
11	1068.8	1074.6	1058.5	1053.7	25	852.5	865.1		
12	1052.8	1058.2	1042.0	1036.9	26	837.5			
13	1036.9	1041.3	1025.6	1020.2	27	822.5			
G(0)	634.1	634.4	635.0	----					

**Table 3:** Vibrational levels and classical turning points for BeF(X^2^∑^+^) radical when *J* = 0 at the MRCI level of theory.

*υ*	*G*(*υ*)/cm^−1^	*R*_min_/nm	*R*_max_/nm	*υ*	*G*(*υ*)/cm^−1^	*R*_min_/nm	*R*_max_/nm
0	634.075	0.13102	0.14423	38	36,940.270	0.10274	0.25274
1	1888.092	0.12696	0.14998	39	37,598.068	0.10253	0.25580
2	3124.450	0.12438	0.15427	40	38,240.767	0.10232	0.25890
3	4343.333	0.12240	0.15798	41	38,868.312	0.10212	0.26207
4	5544.919	0.12077	0.16135	42	39,480.674	0.10193	0.26530
5	6729.378	0.11937	0.16450	43	40,077.768	0.10175	0.26861
6	7896.876	0.11815	0.16751	44	40,659.536	0.10157	0.27199
7	9047.568	0.11705	0.17039	45	41,225.903	0.10139	0.27545
8	10,181.605	0.11606	0.17319	46	41,776.789	0.10123	0.27899
9	11,299.129	0.11516	0.17592	47	42,312.104	0.10107	0.28265
10	12,400.279	0.11432	0.17860	48	42,831.750	0.10092	0.28639
11	13,485.183	0.11355	0.18123	49	43,335.622	0.10077	0.29026
12	14,553.965	0.11283	0.18383	50	43,823.604	0.10063	0.29425
13	15,606.742	0.11216	0.18641	51	44,295.572	0.10049	0.29837
14	16,643.623	0.11153	0.18896	52	44,751.390	0.10037	0.30263
15	17,664.713	0.11094	0.19150	53	45,190.911	0.10024	0.30706
16	18,670.109	0.11037	0.19400	54	45,613.978	0.10020	0.31166
17	19,659.902	0.10984	0.19655	55	46,020.417	0.10010	0.31646
18	20,634.177	0.10934	0.19907	56	46,410.044	0.09990	0.32147
19	21,593.013	0.10886	0.20158	57	46,782.655	0.09980	0.32673
20	22536.484	0.10839	0.20411	58	47138.033	0.09971	0.33226
21	23464.657	0.10796	0.20663	59	47475.938	0.09961	0.33809
22	24377.591	0.10754	0.20916	60	47796.109	0.09953	0.34428
23	25275.345	0.10715	0.21171	61	48098.263	0.09945	0.35088
24	26157.965	0.10677	0.21426	62	48382.086	0.09937	0.35794
25	27025.498	0.10639	0.21683	63	48647.232	0.09930	0.36555
26	27877.980	0.10605	0.21943	64	48893.320	0.09924	0.37383
27	28715.446	0.10571	0.22204	65	49119.923	0.09918	0.38289
28	29537.922	0.10539	0.22467	66	49326.559	0.09912	0.39295
29	30345.429	0.10508	0.22732	67	49512.685	0.09907	0.40426
30	31137.985	0.10478	0.23001	68	49677.674	0.09903	0.41721
31	31915.599	0.10449	0.23272	69	49820.797	0.09899	0.43242
32	32678.277	0.10421	0.23546	70	49941.183	0.09896	0.45089
33	33426.018	0.10394	0.23824	71	50037.765	0.09894	0.47456
34	34158.817	0.10368	0.24106	72	50109.176	0.09892	0.50785
35	34876.662	0.10344	0.24391	73	50153.519	0.09891	0.56546
36	35579.535	0.10319	0.24681	74	50165.999	0.09896	0.65321
37	36267.414	0.10297	0.24975				

**Table 4:** Rotational constants for BeF(X^2^∑^+^) radical.

*υ*	*B_υ/_*cm^−1^			*D_υ/_*cm^−1^		

	This work	Theory^[17]^	Theory^[18]^	This work	Theory^[17]^	Theory^[18]^
0	1.466	1.4640	1.463	7.755	7.865	7.367
1	1.440	1.4471	1.444	7.710	7.888	7.630
2	1.423	1.4297	1.427	7.667	7.827	7.647
3	1.407	1.4132	1.411	7.623	7.820	7.419
4	1.390	1.3971	1.394	7.581	7.817	7.366
5	1.375	1.3808	1.377	7.540	7.728	6.406
6	1.359	1.3641	1.361	7.498	7.669	7.506
7	1.343	1.3475	1.345	7.459	7.695	6.988
8	1.327	1.3310	1.329	7.420	7.630	7.366
9	1.311	1.3146	1.313	7.383	7.605	7.688
10	1.296	1.2984	1.297	7.346	7.555	6.406
11	1.280			7.310		
12	1.265			7.277		
13	1.250			7.245		
14	1.234			7.214		
15	1.219			7.184		
16	1.204			7.157		
17	1.189			7.130		
18	1.174			7.107		
19	1.159			7.084		
20	1.145			7.064		

**Table 5:** The centrifugal distortion constants for the BeF(X^2^∑^+^) radical when *J* = 0.

*υ*	*H_υ_* (×10^11^)/cm^−1^	*L_υ_* (×10^17^)/cm^−1^	*M_υ_* (×10^22^)/cm^−1^	*N_υ_* (×10^27^)/cm^−1^	*O_υ_* (×10^32^)/cm^−1^
0	1.4027100	−4.8671611	1.9911130	−2.8402586	−2.0392494
1	1.4053343	−5.1175272	1.6143796	−3.1990403	−2.2434658
2	1.4053989	−5.3917804	1.2293437	−3.5529674	−2.4676094
3	1.4028724	−5.6889672	0.83591753	−3.9116409	−2.7280207
4	1.3977284	−6.0083544	0.43329623	−4.2808699	−3.0356308
5	1.3899449	−6.3493767	0.020141443	−4.6670931	−3.4018218
6	1.3795027	−6.7116605	−0.40542105	−5.0774004	−3.8395702
7	1.3663844	−7.0950461	−0.84581042	−5.5195056	−4.3611048
8	1.3505725	−7.4996087	−1.3039962	−6.0018194	−4.9798226
9	1.3320486	−7.9256784	−1.7835120	−6.5335708	−5.7128176
10	1.3107919	−8.3738600	−2.2884790	−7.1248038	−6.5756507
11	1.2867778	−8.8450532	−2.8236361	−7.7866333	−7.5878382
12	1.2599765	−9.3404730	−3.3943722	−8.5313740	−8.7747008
13	1.2303516	−9.8616728	−4.0067932	−9.3727447	−10.161238
14	1.1978589	−10.410568	−4.6677733	−10.326299	−11.793475
15	1.1624448	−10.989467	−5.3850704	−11.409132	−13.677149
16	1.1240448	−11.601095	−6.1673928	−12.641287	−15.864538
17	1.0825822	−12.248641	−7.0245304	−14.045587	−18.441352
18	1.0379661	−12.935792	−7.9675652	−15.648035	−21.437787
19	0.99008998	−13.666785	−9.0089937	−17.479025	−24.938023
20	0.93882954	−14.446467	−10.162999	−19.573574	−29.013928

**Table 6:** Comparisons of vibrational levels and molecular constants with experiments and theories calculated for BeF(A^2^Π_r_) radical when *J* = 0.

*υ*	*G*(*υ*)/cm^−1^			*B*_υ_/cm^−1^			*D*_υ_ (×10^6^)/cm^−1^		

	This work	Ref. [14]	Exp. ^*^	This work	Ref. [18]	Exp. [8]	This work	Ref. [18]	Exp. [8]
0	584.86	588	584.1	1.4045	1.4041	1.4115	8.159	8.152	8.40
1	1741.84	1744	1739.1	1.3876	1.3866	1.3939	8.095	8.104	8.26
2	2882.16	2872	2876.6	1.3709	1.3696		8.049	7.953	
3	4005.69	3973	3996.5	1.3545	1.3528		7.981	8.015	
4	5112.92	5047	5098.9	1.3380	1.336		7.926	7.995	
5	6203.86	6097	6183.7	1.3271	1.3192		7.873	7.953	
6	7278.62	7124	7250.9	1.3056	1.3026		7.832	7.884	
7	8337.27	8130	8300.6	1.2897	1.2861		7.777	7.852	
8	9380.07	9117	9332.7	1.2739	1.2695		7.703	7.855	
9	10407.47	10088	10347.3	1.2584	1.2528		7.635	7.856	
10	11419.76	11044	11344.3	1.2430	1.2361		7.603	7.831	
11	12416.79	12925	13285.6	1.2276			7.611		
12	13398.11	13855	14229.9	1.1212			7.634		
13	14363.21	14779	15156.7	1.1961			7.603		
14	15312.16			1.1807			7.451		
15	16246.14			1.166			7.162		
16	17167.19			1.1526			6.895		
17	18076.98			1.1397			6.919		
18	18974.86			1.1257			7.418		
19	19275.90			2.3327			6.9808		
20	19313.93			2.0731			2.9969		

* Taken from the reference in Reference [14].

**Table 7:** Vibrational levels and molecular constants for the B^2^∑^+^ state of BeF radical.

*υ*	*G*(*υ*)/cm^−1^	*B_υ_*/cm^−1^	*D_υ_*(×10^6^)/cm^−1^
0	672.36	1.5451	8.263
1	1997.79	1.5248	8.310
2	3297.21	1.5042	8.533
3	3565.79	0.3669	1.304
4	3953.60	0.3715	1.377
5	4342.89	0.3757	1.428
6	4570.02	1.4833	8.444
7	4733.41	0.3795	1.483
8	5124.94	0.3832	1.533
9	5517.25	0.3866	1.584
10	5815.89	1.4621	8.580
11	5910.18	0.3898	1.632
12	6303.56	0.3928	1.686
13	6697.25	0.3957	1.741
14	7033.16	1.4399	8.771
15	7091.10	0.3984	1.791
16	7484.95	0.4010	1.849
17	7878.67	0.4034	1.909
18	8220.25	1.4176	8.725
19	8272.16	0.4057	2.001
20	8665.01	0.4079	2.056
